# Mechanical Modeling of Tube Bending Considering Elastoplastic Evolution of Tube Cross-Section

**DOI:** 10.3390/ma15113997

**Published:** 2022-06-03

**Authors:** Zongcai Zhang, Jianjun Wu, Xinliang Xu, Zekun Yang, Wei Wu, Long Liu

**Affiliations:** 1 School of Mechanical Engineering, Northwestern Polytechnical University, Xi’an 710072, China or zhzoci@mail.nwpu.edu.cn (Z.Z.); y17381591816@163.com (Z.Y.); losliu@mail.nwpu.edu.cn (L.L.); 2 AVIC Manufacturing Technology Institute, Beijing 100024, China; xuxinls@163.com (X.X.); wuweifiles@163.com (W.W.)

**Keywords:** free bending forming, section deformation mechanism, stress superposition, aluminum alloy tube, mechanical model

## Abstract

Aluminum alloy tubes are widely used in various industries because of their excellent performance. Up to now, when the tube is bent, the elastoplastic deformation evolution mechanism of the cross-section has not been clear, and no direct analytical proof has been found. In this paper, based on the bilinear material model assumption, a new mechanical model of tube plane bending deformation is constructed. The analytical model can describe in detail the evolution mechanism of elastic–plastic deformation on the cross-section of the tube after bending deformation, the position of the elastic–plastic boundary, the position of the radius of the strain neutral layer, and the relationship between the bending moment over the section and the bending radius. According to this model, the deformation law of the tube cross-section during bending is elucidated. The results are as follows: (1) the deformation evolution of the cross-section of the bending deformed tube calculated by the analytical model is in good agreement with the finite element model (FEM) of pure bending. (2) By comparing the results of the analytical model with FEM results, and the processing test of the self-designed four-axis free bending forming tube bender, the bending moments are in good agreement. (3) Compared with the bending moments calculated by several other analytical models of tube bending, this model has a relatively small deviation value.

## 1. Introduction

In aviation, aerospace, ship building, automobile production, and other industrial systems, a large number of circular tubes are often used to transmit fuel, hydraulic control medium, air pressure control medium, etc., to achieve the long-distance transmission of working medium [1]. In the existing literature, a large amount of research can be found on the theoretical analysis of tube bending deformation. Al-Qureshi and Russo [2,3] presented a theoretical analysis of the elastic–plastic bending of the thin-walled tube, and by establishing approximate equations, a quantitative method for predicting the springback behavior and residual stress distributions was provided. At the same time, Tang [4] developed practical formulae to explain the elastic–plastic deformation behavior of thin-walled tubes, such as stresses, wall thickness change, shrinking rate at the tube section, deviation of neutral axis, and bending moment. Lu Shiqiang et al. [5] also revised this analytical model and, based on plane strain assumption and exponent hardening law, investigated the plastic deformation in tube bending. Some similar theoretical formulae were developed to explain the phenomena in tube bending and were validated by the springback experiment. Megharbel et al. [6] performed a theoretical analysis on the elastic–plastic bending of square and circular pipes using the constitutive equation of the power exponent reinforced material and provided an analytical method in the form of an analytical equation to predict the moment forming a tube section with a specific radius of curvature. Using the plate bending theory, Daxin et al. [7] deduced the approximate calculation formula of the radius of the strain neutral layer and the thinning amount of the outer tube wall thickness and further revealed the deformation mechanism of tube bending. However, because the influence of deformation perpendicular to the bending plane direction was ignored, and the material performance parameters were not considered, the calculated value was too large and thus needs further correction. Using the neutral layer offset formula established by Daxin, Zhan et al. established an analytical model of pipe bending springback [8]. Zhu et al. [9] developed a theoretical analysis model based on this model that can calculate the springback angle of rectangular H96 tubes. Cheng et al. recently improved the analytical model to allow the mechanical analysis of thin-walled tubes with mandrel support or welds [10,11]. Meanwhile, Li et al. established a hybrid analytical–numerical model based on axial force balance to analyze the neutral layer shifting (NLS) phenomenon in the process of tube bending and thus proposed an innovative process to improve bend formability by analyzing the equilibrium conditions of bending moment and force in the process of tube bending [12]. They also constructed another method for calculating the radius of the neutral layer [13], and this method was used by Fang Jun et al. [14] to analyze the thinning behavior of the 0Cr21Ni6Mn9N tube. Moreover, to improve the tube geometry, mechanical properties, and formability, an integrated machining strategy was proposed by Ma et al., which integrates cold bending operations and heat treatment, and the expected dimensional accuracy and mechanical properties were achieved [15]. However, none of these analytical models took into account the elastoplastic evolution of the tube cross-section when the tube was bent.

On the other hand, in terms of tube forming technology, three-dimensional free bending forming technology has become another rapidly developing metal tube forming technology, compared with conventional tube bending techniques, such as roll-draw bending, and roll-pull bending. It can realize the continuous multi-bending and one-time forming of the tube without changing the die assembly, and the forming effect is quite good [16,17]. The basic principle of a torque superposed spatial bending method was proposed by Brosius et al. [18] and Staupendahl et al. [19,20]. Based on this principle and the theory of elastic–plastic mechanics, Hudovernik et al. [21] established an analytical model for the spatial bending of square tubes and verified the validity of the analytical model by comparing the forming process parameters obtained by numerical simulation, experiment, and analytical calculation. However, this model assumed that the strain on the section was balanced, so the error was relatively large. Staupendahl [22] improved this model and proposed an analytical model in which the strain decreases linearly along the edge to the central layer. Similarly, in the aspect of the free bending forming of circular tubes, Zhang et al. [23,24] and Wu et al. [25], among others, established a mechanical analytical model for the combined deformation of circular tubes in space by bending and twisting and studied the elastic–plastic deformation mechanism of the cross-section. Bending and torsional deformation of composite pipes may become a new research focus in the future. Jonnalagadda et al. [26] developed a straightforward analytical model of a composite tube subjected to bending and torsion. This analytical model, however, ignored the stress–strain variation in the thickness direction of the tube. In summary, the analytical models proposed in the preceding papers were only roughly proven by varying the tube bending radius values in the numerical model and sample test, which were not validated by numerical simulation of the elastic–plastic deformation evolution of the tube section.

In this paper, based on the Mises yield criterion, the law of full quantity, and the principle of stress superposition, the mechanical model of the plane bending deformation of the tube was established by using an aluminum alloy bilinear material model. This analytical model was used to analyze the elastic–plastic evolution of the tube cross-section, the position of the elastic–plastic boundary, and the radius of the strain neutral layer. Furthermore, the relationship between the bending moment of the section and the bending radius was also obtained. Moreover, to verify the accuracy and validity of the analytical model and further reveal the mechanical mechanism of bending forming of the tube, the mechanical model was compared with the simplified finite element model (SFEM) of pure bending forming of the tube, the FEM of free bending forming of the tube, and the physical test of free bending forming of the tube carried out by the self-designed four-axis free bending forming equipment; the results of the bending moment were in good agreement.

## 2. Mechanical Model of Free Bending of Tube

### 2.1. Sectional Geometry and Pure Bending Deformation of the Tube

**Figure 1:**
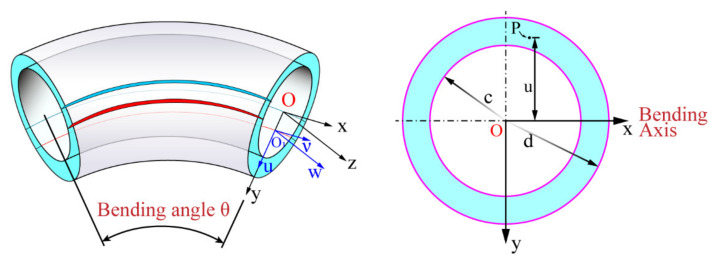
Schematic diagram of cross-sectional shape and pure bending forming of the tube.

Figure 1 shows the schematic diagram of the cross-sectional shape and pure bending forming of the tube. The center of the tube on the cross-section is taken as the origin O of the coordinate system, and the plane where the cross-section is located and the pure bending plane are taken as the coordinate plane to establish the Cartesian coordinate system Oxyz. In this coordinate system, the longitudinal section Oyz of the tube is the pure bending plane; the *x*-axis is the bending rotation axis of the cross-section; and the *z*-axis passes through the geometric centroid of the tube of all cross-sections and is perpendicular to the cross-section. The outer radius and inner radius of the tube are d and c, respectively.

### 2.2. Principle of Three-Axis Free Bending Forming Technology and Bending Moment Calculation

**Figure 2:**
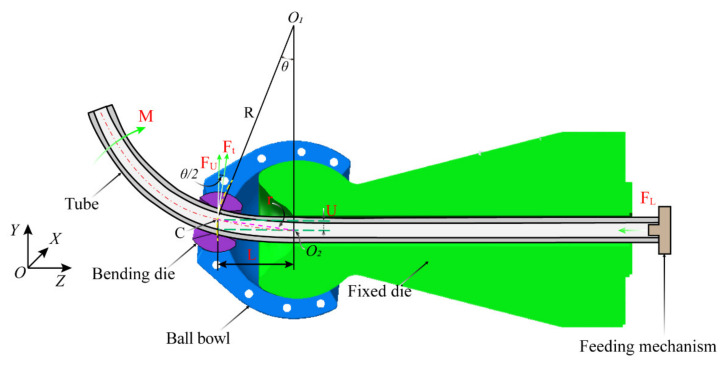
Schematic diagram of free bending forming device.

As shown in Figure 2, a typical three-axis free bending forming system is mainly composed of five parts: the tube, bending die, ball bowl, fixed die, and feeding mechanism. During the free bending process of the tube, the feeding mechanism continuously pushes the tube through the fixed die and the bending die along the *z*-axis under the driving action of the z-direction drive motor. At the same time, the ball bowl rotates around the center point $O_{2}$ of the fixed die outlet under the driving action of the *x*- and *y*-axis motors; the bending die is embedded at the outlet of the ball bowl to achieve dynamic contact with the tube and rotate around the fixed die. Here, the radius of revolution of the bending die is r. The bending die, the ball bowl, and the fixed die all have spherical contacts. The rotation and offset of the bending die promote the bending of the tube. R is the bending radius of the geometric center layer after the free bending deformation of the tube, and $O_{1}$ is the bending arc center of the tube. During the forming process, the tube is subjected to the combined action of the force $F_{t}$ from the bending die perpendicular to the axis of the tube and the force $F_{L}$ along the z-direction from the feeding mechanism, and the combined action of $F_{t}$ and $F_{L}$ causes the tube to bend. Hence, according to the geometric relationship, the *y*-axis direction component $F_{U}$ of $F_{t}$ can be expressed as

(1) $$F_{U}=F_{T}\cos\left(\frac{\theta }{2}\right)$$

It is assumed that the hole center point C of the bending die is the force application point for the tube to bear the bending moment, so the bending moment M is calculated as Equation (2):

(2) $$M=F_{t}\;r\;{\left(\cos\left(\frac{\theta }{2}\right)\right)}^{2}+F_{L}\;r\;\sin\left(\frac{\theta }{2}\right)$$

### 2.3. Basic Assumptions

In order to facilitate the study of section deformation of the tube and simplify the derivation process of formula, the following basic assumptions are made according to the basic principle of elastic–plastic bending theory.

(1) Unidirectional stress–strain assumption: it is assumed that each fiber of the profile tube wall is in the stress–strain state of unidirectional tension or compression during the free bending deformation of the tube.
(2) Plane strain assumption: it is assumed that the cross-section of the tube is always plane before and after free bending deformation, without warping deformation, and the position of the geometric center point of the section does not change.
(3) Bilinear material model assumption: it is assumed that the tube is a homogeneous material, a continuous elastic–plastic deformation body, and the stress–strain relationship under unidirectional loading is

(3) $$\sigma =\left\{\begin{array}{cc} \begin{array}{l} \varepsilon E\\ \sigma_{\begin{array}{c} Y \end{array}}+\left(\varepsilon -\varepsilon_{Y}\right)D \end{array} & \begin{array}{c} \varepsilon \leq \varepsilon_{Y}\\ \varepsilon >\varepsilon_{Y} \end{array} \end{array}\right.$$

where $\sigma_{\begin{array}{c} y \end{array}}=\varepsilon_{Y}E$.

### 2.4. Analytical Model of Mechanics

After free bending deformation of the tube, the strain neutral layer moves to the inner layer, and the strain neutral layer does not coincide with the geometric center layer. In the bending plane, it is assumed that the coordinate system at the strain neutral layer is $O_{1}uvw$ (illustrated in Figure 1), and the coordinate system at the geometric center layer remains unchanged and is still Oxyz.

**Figure 3:**
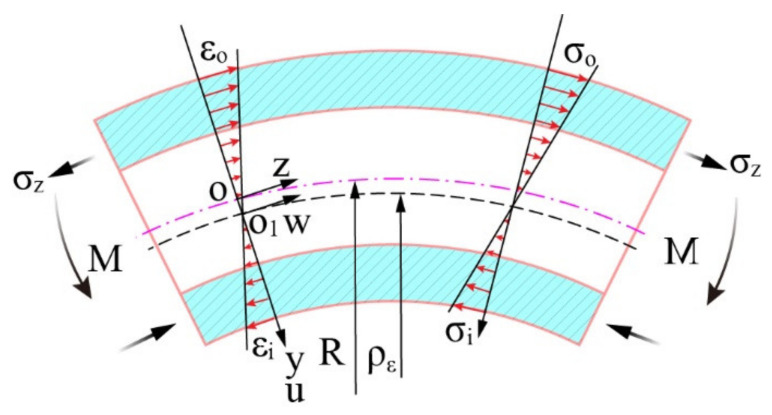
Fully elastic deformation stage.

**Figure 4:**
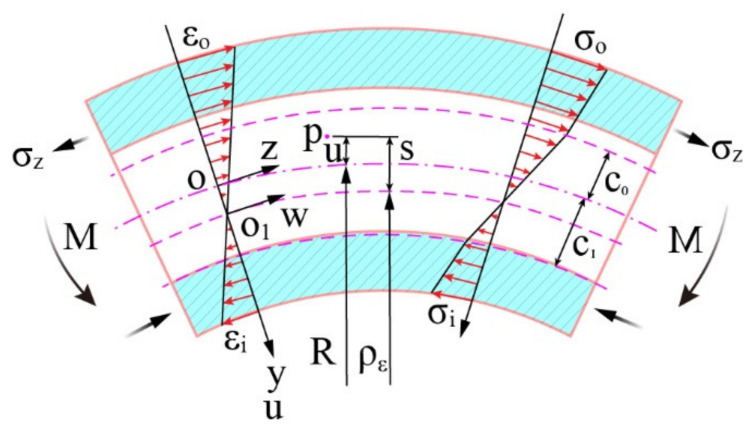
Elastic–plastic deformation stage.

Under a certain bending radius R, when different external bending moments M are applied externally, the distribution of stress and strain on the cross-section of the tube is different after bending deformation. Therefore, according to the different conditions of the stress–strain distribution state of the section, the stress–strain distribution state of the section can be divided into the fully elastic deformation stage (shown in Figure 3) and the elastic–plastic deformation stage (depicted in Figure 4).

In the coordinate system$\;O_{1}uw$ where the strain neutral layer is located as shown in Figure 4, the tangential engineering strain at a point P on the section can be expressed as

(4) $$\varepsilon =\frac{s}{\rho_{\varepsilon }}\;\mathrm{for}\;R-\rho_{\varepsilon }-d\leq s\leq R-\rho_{\varepsilon }+d$$

There is a coordinate conversion relationship between the coordinate system $O_{1}uw$ where the strain neutral layer is located and the coordinate system *Oyz* where the geometric center layer is located: $u=s+\left(R-\rho_{\varepsilon }\right)$. Zhai Ruixue et al. also used this formula to conduct the springback analysis of rectangular profiles in tension bending [27]. Therefore, the expression of the tangential strain of the section in the coordinate system Oyz where the geometric center layer is located can be found as

(5) $$\varepsilon =\frac{\left(R-\rho_{\varepsilon }\right)-u}{\rho_{\varepsilon }}\;\mathrm{for}-d\leq u\leq +d$$

According to the assumption of the bilinear material model, the expression of the total tangential stress on the section shown in Figure 4 under the elastic–plastic deformation state is calculated by

(6) $$\sigma =\left\{\begin{array}{cc} \begin{array}{l} -\sigma_{Y}+\left(\varepsilon +\varepsilon_{Y}\right)D\\ \varepsilon E\\ \sigma_{Y}+\left(\varepsilon -\varepsilon_{Y}\right)D \end{array} & \begin{array}{c} c_{1}\leq u\leq d\\ c_{0}\leq u\leq c_{1}\\ -d\leq u\leq c_{0} \end{array} \end{array}\right.$$

The following relationship can be obtained by substituting Equation (5) into Equation (6):

(7) $$\sigma =\left\{\begin{array}{ll} -\sigma_{Y}+\left(\frac{\left(R-\rho_{\varepsilon }\right)-u}{\rho_{\varepsilon }}+\varepsilon_{Y}\right)D & c_{1}\leq u\leq d\\ \frac{\left(R-\rho_{\varepsilon }\right)-u}{\rho_{\varepsilon }}E & c_{0}\leq u\leq c_{1}\\ \sigma_{Y}+\left(\frac{\left(R-\rho_{\varepsilon }\right)-u}{\rho_{\varepsilon }}-\varepsilon_{Y}\right)D & -d\leq u\leq c_{0} \end{array}\right.$$

**Figure 5:**
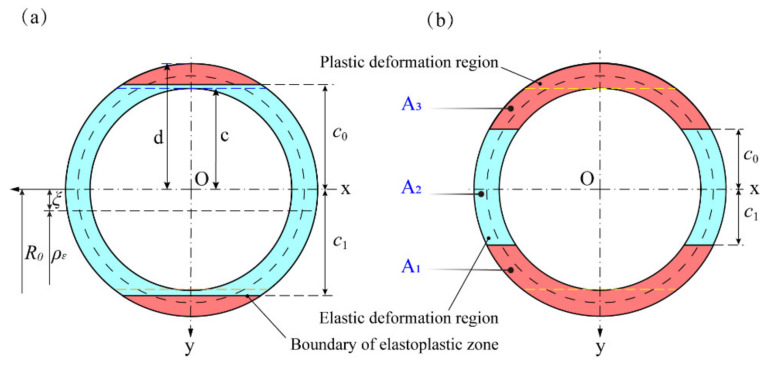
Evolution of the elastic-plastic deformation region of the cross-section. (**a**) The elastoplastic boundary lies between inner radius c and outer radius d along the y-direction; (**b**) The elastoplastic boundary lies between geometric center layer and inner radius c along the y-direction.

In the elastic–plastic deformation stage, the cross-section of the tube can be divided into three deformation regions, namely the outer tensile plastic deformation area, the middle elastic deformation area, and the inner compressive plastic deformation area. Figure 5a shows that the plastic deformation starts from the outer radius of the section along the y-direction and gradually expands to the inner radius; during this deformation process, the elastic–plastic boundary line lies between c and d. As the bending degree of the tube increases, the boundary line of the elastic–plastic region expands further inward and moves between the strain-neutral layer and the inner radius c, as shown in Figure 5b. Following this, the elastoplastic deformation evolution of the cross-section shown in Figure 5 is analytically modeled, and the position of the elastoplastic boundary line, the position of the strain-neutral layer, and the applied bending moment on the cross-section in the corresponding deformation state are obtained.

As depicted in Figure 5, on any cross-section, the areas of the compressive plastic deformation zone, the elastic deformation zone, and the tensile plastic deformation zone are $A_{1}$, $A_{2}$ and $A_{3}$, respectively, and the static moments of these to the *x*-axis are $S_{1}$, $S_{2}$ and $S_{3}$, respectively. Similarly, it is assumed that the moments of inertia about the *x*-axis are $I_{1}$, $I_{2}$ and $I_{3}$, respectively.

Case (a): $-d\leq c_{0}\leq -c$ and $c\leq c_{1}\leq d$

As illustrated in Figure 5a, the area of the tensile plastic deformation zone $A_{3}$ in the tube can be obtained by Equation (8).

(8) $$A_{3}=\int_{-d}^{c_{0}}2\sqrt{d^{2}-y^{2}}dy=2d^{2}\left(\frac{1}{2}t+\frac{1}{4}\sin2t\right)\left|\begin{array}{c} t_{2}\\ t_{1} \end{array}\right.$$

where $t_{1}=\sin^{-1}\frac{-d}{d}$, $t_{2}=\sin^{-1}\frac{c_{0}}{d}$.

Similarly, the area of the compressive plastic deformation zone $A_{1}$ can be calculated by Equation (9).

(9) $$A_{1}=\int_{c_{1}}^{d}2\sqrt{d^{2}-y^{2}}dy=2d^{2}\left(\frac{1}{2}t+\frac{1}{4}\sin2t\right)\left|\begin{array}{c} t_{4}\\ t_{3} \end{array}\right.$$

where $t_{3}=\sin^{-1}\frac{c_{1}}{d}$, $t_{4}=\sin^{-1}\frac{d}{d}$.

Then, the area of the elastic deformation zone $A_{2}$ of the tube cross-section can be obtained:

(10) $$A_{2}=\pi \left(d^{2}-c^{2}\right)-2d^{2}\left(\left(\frac{1}{2}t+\frac{1}{4}\sin2t\right)\left|\begin{array}{c} t_{2}\\ t_{1} \end{array}\right.+\left(\frac{1}{2}t+\frac{1}{4}\sin2t\right)\left|\begin{array}{c} t_{4}\\ t_{3} \end{array}\right.\right)$$

where $t_{1}=\sin^{-1}\frac{-d}{d}$, $t_{2}=\sin^{-1}\frac{c_{0}}{d}$, $t_{3}=\sin^{-1}\frac{c_{1}}{d}$, $t_{4}=\sin^{-1}\frac{d}{d}$.

It can be seen from Figure 5a that the static moment in the tensile plastic deformation zone can be obtained by Equation (11):

(11) $$S_{3}=\int_{-d}^{c_{0}}2y\sqrt{d^{2}-y^{2}}dy=\frac{-2d^{3}}{3}{\left(\cos t\right)}^{3}\left|\begin{array}{c} t_{2}\\ t_{1} \end{array}\right.$$

where $t_{1}=\sin^{-1}\frac{-d}{d}$, $t_{2}=\sin^{-1}\frac{c_{0}}{d}$.

Similarly, the static moment in the compressive plastic deformation zone can be obtained by Equation (12):

(12) $$S_{1}=\int_{c_{1}}^{d}2y\sqrt{d^{2}-y^{2}}dy=\frac{-2d^{3}}{3}{\left(\cos t\right)}^{3}\left|\begin{array}{c} t_{4}\\ t_{3} \end{array}\right.$$

where $t_{3}=\sin^{-1}\frac{c_{1}}{d},\;\;t_{4}=\sin^{-1}\frac{d}{d}$.

The static moment in the elastic deformation zone can be expressed as $S_{2}=-\left(S_{1}+S_{3}\right)$, and thus Equation (13) can be obtained by incorporating Equations (11) and (12) as follows:

(13) $$S_{2}=\frac{2d^{3}}{3}{\left(\cos t\right)}^{3}\left|\begin{array}{c} t_{2}\\ t_{1} \end{array}\right.+\frac{2d^{3}}{3}{\left(\cos t\right)}^{3}\left|\begin{array}{c} t_{4}\\ t_{3} \end{array}\right.$$

where $t_{1}=\sin^{-1}\frac{-d}{d}$, $t_{2}=\sin^{-1}\frac{c_{0}}{d}$, $t_{3}=\sin^{-1}\frac{c_{1}}{d}$, $t_{4}=\sin^{-1}\frac{d}{d}$.

Further, as depicted in Figure 5a, the moment of inertia in the tensile plastic deformation zone can be obtained by Equation (14):

(14) $$I_{3}=\int_{-d}^{c_{0}}2y^{2}\sqrt{d^{2}-y^{2}}dy=\frac{d^{4}}{2}\left(\frac{1}{2}t-\frac{1}{8}\sin4t\right)\left|\begin{array}{c} t_{2}\\ t_{1} \end{array}\right.$$

where $t_{1}=\sin^{-1}\frac{-d}{d}$, $t_{2}=\sin^{-1}\frac{c_{0}}{d}$.

Similarly, the moment of inertia in the compressive plastic deformation zone can be obtained by Equation (15):

(15) $$I_{1}=\int_{c_{1}}^{d}2y^{2}\sqrt{d^{2}-y^{2}}dy=\frac{d^{4}}{2}\left(\frac{1}{2}t-\frac{1}{8}\sin4t\right)\left|\begin{array}{c} t_{4}\\ t_{3} \end{array}\right.\;$$

where $t_{3}=\sin^{-1}\frac{c_{1}}{d}$, $t_{4}=\sin^{-1}\frac{d}{d}$.

Likewise, according to the mechanics of materials, the moment of inertia of the circular tube section shown in Figure 1 to the central axis can be expressed as

(16) $$I_{x}=\frac{\pi }{4}\left(d^{4}-c^{4}\right)$$

the moment of inertia in the elastic deformation zone is $I_{2}=I_{x}-I_{1}-I_{3}$; that is,

(17) $$\begin{array}{c} I_{2}=\frac{\pi }{4}\left(d^{4}-c^{4}\right)-\frac{d^{4}}{2}\left(\frac{1}{2}t-\frac{1}{8}\sin4t\right)\left|\begin{array}{c} t_{2}\\ t_{1} \end{array}\right.-\frac{d^{4}}{2}\left(\frac{1}{2}t-\frac{1}{8}\sin4t\right)\left|\begin{array}{c} t_{4}\\ t_{3} \end{array}\right. \end{array}$$

where $t_{1}=\sin^{-1}\frac{-d}{d}$, $t_{2}=\sin^{-1}\frac{c_{0}}{d}$, $t_{3}=\sin^{-1}\frac{c_{1}}{d}$,$\;t_{4}=\sin^{-1}\frac{d}{d}$.

Case (b): $-c<c_{0}\leq 0$ and $0<c_{1}\leq c$

As illustrated in Figure 5b, the area of the tensile plastic deformation zone can be obtained by Equation (18):

(18) $$\begin{array}{l} A_{3}=\int_{-d}^{-c}2\sqrt{d^{2}-y^{2}}dy+\int_{-c}^{c_{0}}2\left(\sqrt{d^{2}-y^{2}}-\sqrt{c^{2}-y^{2}}\right)dy\\ \;\;\;\;\;=2d^{2}\left(\left(\frac{1}{2}t+\frac{1}{4}\sin2t\right)\left|\begin{array}{c} t2\\ t1 \end{array}\right.+\left(\frac{1}{2}t+\frac{1}{4}\sin2t\right)\left|\begin{array}{c} t_{4}\\ t_{3} \end{array}\right.\right)-2c^{2}\left(\frac{1}{2}t+\frac{1}{4}\sin2t\right)\left|\begin{array}{c} t_{6}\\ t_{5} \end{array}\right. \end{array}$$

where $t_{1}=\sin^{-1}\frac{-d}{d}$, $t_{2}=\sin^{-1}\frac{-c}{d}$, $t_{3}=\sin^{-1}\frac{-c}{d}$, $t_{4}=\sin^{-1}\frac{c_{0}}{d}$, $t_{5}=\sin^{-1}\frac{-c}{c}$, $t_{6}=\sin^{-1}\frac{c_{0}}{c}$.

Similarly, As described in Figure 5b, the area of the compressive plastic deformation zone can be calculated by Equation (19):

(19) $$\begin{array}{l} A_{1}=\int_{c_{1}}^{c}2\left(\sqrt{d^{2}-y^{2}}-\sqrt{c^{2}-y^{2}}\right)dy+\int_{c}^{d}2\sqrt{d^{2}-y^{2}}dy\\ \;\;\;\;\;=2d^{2}\left(\left(\frac{1}{2}t+\frac{1}{4}\sin2t\right)\left|\begin{array}{c} t_{2}^{'}\\ t_{1}^{'} \end{array}\right.+\left(\frac{1}{2}t+\frac{1}{4}\sin2t\right)\left|\begin{array}{c} t_{4}^{'}\\ t_{3}^{'} \end{array}\right.\right)-2c^{2}\left(\frac{1}{2}t+\frac{1}{4}\sin2t\right)\left|\begin{array}{c} t_{6}^{'}\\ t_{5}^{'} \end{array}\right. \end{array}$$

where $t_{1}^{'}=\sin^{-1}\frac{c}{d}$, $t2^{'}=\sin^{-1}\frac{d}{d}$, $t3^{'}=\sin^{-1}\frac{c_{1}}{d}$, $t4^{'}=\sin^{-1}\frac{c}{d}$, $t5^{'}=\sin^{-1}\frac{c_{1}}{c}$, $t6^{'}=\sin^{-1}\frac{c}{c}$.

Then the area of the elastic deformation zone $A_{2}$, as shown in Figure 5b, can be expressed as Equation (20):

(20) $$\begin{array}{cc} A_{2} & =\pi \left(d^{2}-c^{2}\right)\\ & +2c^{2}\left(\left(\frac{1}{2}t+\frac{1}{4}\sin2t\right)\left|\begin{array}{c} t_{6}\\ t_{5} \end{array}\right.+\left(\frac{1}{2}t+\frac{1}{4}\sin2t\right)\left|\begin{array}{c} t_{6}^{'}\\ t_{5}^{'} \end{array}\right.\right)\\ & \;-2d^{2}\left[\left(\frac{1}{2}t+\frac{1}{4}\sin2t\right)\left|\begin{array}{c} t_{2}\\ t_{1} \end{array}\right.+\left(\frac{1}{2}t+\frac{1}{4}\sin2t\right)\left|\begin{array}{c} t_{4}\\ t_{3} \end{array}\right.\right]\\ & -2d^{2}\left[\left(\frac{1}{2}t+\frac{1}{4}\sin2t\right)\left|\begin{array}{c} t_{2}^{'}\\ t_{1}^{'} \end{array}\right.+\left(\frac{1}{2}t+\frac{1}{4}\sin2t\right)\left|\begin{array}{c} t_{4}^{'}\\ t_{3}^{'} \end{array}\right.\right] \end{array}$$

where $t_{1}=\sin^{-1}\frac{-d}{d}$, $t_{2}=\sin^{-1}\frac{-c}{d}$, $t_{3}=\sin^{-1}\frac{-c}{d}$, $t_{4}=\sin^{-1}\frac{c_{0}}{d}$, $t_{5}=\sin^{-1}\frac{-c}{c}$, $t_{6}=\sin^{-1}\frac{c_{0}}{c}$, $t_{1}^{'}=\sin^{-1}\frac{c}{d}$, $t_{2}^{'}=\sin^{-1}\frac{d}{d}$, $t_{3}^{'}=\sin^{-1}\frac{c_{1}}{d}$, $t_{4}^{'}=\sin^{-1}\frac{c}{d}$, $t_{5}^{'}=\sin^{-1}\frac{c_{1}}{c}$, $t_{6}^{'}=\sin^{-1}\frac{c}{c}$.

Moreover, the static moment in the tensile plastic deformation zone can be obtained by Equation (21):

(21) $$\begin{array}{cc} S_{3} & =\int_{-d}^{-c}2y\sqrt{d^{2}-y^{2}}dy+\int_{-c}^{c_{0}}2y\left(\sqrt{d^{2}-y^{2}}-\sqrt{c^{2}-y^{2}}\right)dy\\ & =\frac{2c^{3}}{3}{\left(\cos t\right)}^{3}\left|\begin{array}{c} t_{6}\\ t_{5} \end{array}\right.-\frac{2d^{3}}{3}{\left(\cos t\right)}^{3}\left|\begin{array}{c} t_{2}\\ t_{1} \end{array}\right.-\frac{2d^{3}}{3}{\left(\cos t\right)}^{3}\left|\begin{array}{c} t_{4}\\ t_{3} \end{array}\right. \end{array}$$

where$\;t_{1}=\sin^{-1}\frac{-d}{d}$, $t_{2}=\sin^{-1}\frac{-c}{d}$, $t_{3}=\sin^{-1}\frac{-c}{d}$, $t_{4}=\sin^{-1}\frac{c_{0}}{d}$, $t_{5}=\sin^{-1}\frac{-c}{c}$, $t_{6}=\sin^{-1}\frac{c_{0}}{c}$.

Similarly, the static moment in the compressive plastic deformation zone can be obtained by Equation (22):

(22) $$\begin{array}{cc} S_{1} & =\int_{c_{1}}^{c}2y\left(\sqrt{d^{2}-y^{2}}-\sqrt{c^{2}-y^{2}}\right)dy+\int_{c}^{d}2y\sqrt{d^{2}-y^{2}}dy\\ & =\frac{2c^{3}}{3}{\left(\cos t\right)}^{3}\left|\begin{array}{c} t_{6}^{'}\\ t_{5}^{'} \end{array}\right.-\frac{2d^{3}}{3}{\left(\cos t\right)}^{3}\left|\begin{array}{c} t_{4}^{'}\\ t_{3}^{'} \end{array}\right.-\frac{2d^{3}}{3}{\left(\cos t\right)}^{3}\left|\begin{array}{c} t_{2}^{'}\\ t_{1}^{'} \end{array}\right. \end{array}$$

where $t_{1}^{'}=\sin^{-1}\frac{c}{d}$, $t_{2}^{'}=\sin^{-1}\frac{d}{d}$, $t_{3}^{'}=\sin^{-1}\frac{c_{1}}{d}$, $t_{4}^{'}=\sin^{-1}\frac{c}{d}$, $t_{5}^{'}=\sin^{-1}\frac{c_{1}}{c}$, $t_{6}^{'}=\sin^{-1}\frac{c}{c}$.

The static moment in the elastic deformation zone can be expressed as $S_{2}=-\left(S_{1}+S_{3}\right)$. Thus, Equation (23) can be obtained by incorporating Equations (21) and (22), as follows:

(23) $$S_{2}=\frac{2d^{3}}{3}\left({\left(\cos t\right)}^{3}\left|\begin{array}{c} t_{2}\\ t_{1} \end{array}\right.+{\left(\cos t\right)}^{3}\left|\begin{array}{c} t_{2}^{'}\\ t_{1}^{'} \end{array}\right.\right)+\frac{2d^{3}}{3}\left({\left(\cos t\right)}^{3}\left|\begin{array}{c} t_{4}\\ t_{3} \end{array}\right.+{\left(\cos t\right)}^{3}\left|\begin{array}{c} t_{4}^{'}\\ t_{3}^{'} \end{array}\right.\right)-\frac{2c^{3}}{3}\left({\left(\cos t\right)}^{3}\left|\begin{array}{c} t_{6}\\ t_{5} \end{array}\right.+{\left(\cos t\right)}^{3}\left|\begin{array}{c} t_{6}^{'}\\ t_{5}^{'} \end{array}\right.\right)$$

where $t_{1}=\sin^{-1}\frac{-d}{d}$, $t_{2}=\sin^{-1}\frac{-c}{d}$, $t_{3}=\sin^{-1}\frac{-c}{d}$, $t_{4}=\sin^{-1}\frac{c_{0}}{d}$, $t_{5}=\sin^{-1}\frac{-c}{c}$, $t_{6}=\sin^{-1}\frac{c_{0}}{c}$, $t_{1}^{'}=\sin^{-1}\frac{c}{d}$, $t_{2}^{'}=\sin^{-1}\frac{d}{d}$, $t_{3}^{'}=\sin^{-1}\frac{c_{1}}{d}$, $t_{4}^{'}=\sin^{-1}\frac{c}{d}$, $t_{5}^{'}=\sin^{-1}\frac{c_{1}}{c}$, $t_{6}^{'}=\sin^{-1}\frac{c}{c}$.

As illustrated in Figure 5b, in order to facilitate the integration, the inertia moment of the elastic deformation zone needs to be divided into two parts to calculate separately, namely the inertia moment $I_{2}^{\prime }$ of the lower half of the *x*-axis and the inertia moment $I_{2}^{\prime \prime }\;$of the upper half of the *x*-axis. $I_{2}^{'}$ and $I_{2}^{\prime \prime }$ can be obtained by Equations (24) and (25), respectively.

(24) $$\begin{array}{rr} I_{2}^{\prime } & =\int_{c_{0}}^{0}2y^{2}\left(\sqrt{d^{2}-y^{2}}-\sqrt{c^{2}-y^{2}}\right)dy\\ & =\frac{d^{4}}{2}\left(\frac{1}{2}t-\frac{1}{8}\sin4t\right)\left|\begin{array}{c} t_{2}\\ t_{1} \end{array}\right.-\frac{c^{4}}{2}\left(\frac{1}{2}t-\frac{1}{8}\sin4t\right)\left|\begin{array}{c} t_{4}\\ t_{3} \end{array}\right. \end{array}$$

where $t_{1}=\sin^{-1}\frac{c_{0}}{d}$, $t_{2}=0$, $t_{3}=\sin^{-1}\frac{c_{0}}{c}$, $t_{4}=0$.

(25) $$\begin{array}{rr} I_{2}^{\prime \prime } & =\int_{0}^{c_{1}}2y^{2}\left(\sqrt{d^{2}-y^{2}}-\sqrt{c^{2}-y^{2}}\right)dy\\ & =\frac{d^{4}}{2}\left(\frac{1}{2}t-\frac{1}{8}\sin4t\right)\left|\begin{array}{c} t_{2}\\ t_{1} \end{array}\right.-\frac{c^{4}}{2}\left(\frac{1}{2}t-\frac{1}{8}\sin4t\right)\left|\begin{array}{c} t_{4}\\ t_{3} \end{array}\right. \end{array}$$

where $t_{1}=0$, $t_{2}=\sin^{-1}\frac{c_{1}}{d}$, $t_{3}=0$, $t_{4}=\sin^{-1}\frac{c_{1}}{c}$.

Furthermore, using material mechanics, the moment of inertia $I_{3}\;$in the tensile plastic deformation zone can be obtained by Equation (26):

(26) $$I_{3}=\frac{\pi }{8}\left(d^{4}-c^{4}\right)-\frac{d^{4}}{2}\left(\frac{1}{2}t-\frac{1}{8}\sin4t\right)\left|\begin{array}{c} t_{2}\\ t_{1} \end{array}\right.+\frac{c^{4}}{2}\left(\frac{1}{2}t-\frac{1}{8}\sin4t\right)\left|\begin{array}{c} t_{4}\\ t_{3} \end{array}\right.$$

where $t_{1}=\sin^{-1}\frac{c_{0}}{d}$, $t_{2}=0$, $t_{3}=\sin^{-1}\frac{c_{0}}{c}$, $t_{4}=0$.

Likewise, the moment of inertia in the compressive plastic deformation zone can be acquired by Equation (27):

(27) $$I_{1}=\frac{\pi }{8}\left(d^{4}-c^{4}\right)-\frac{d^{4}}{2}\left(\frac{1}{2}t-\frac{1}{8}\sin4t\right)\left|\begin{array}{c} t_{2}\\ t_{1} \end{array}\right.+\frac{c^{4}}{2}\left(\frac{1}{2}t-\frac{1}{8}\sin4t\right)\left|\begin{array}{c} t_{4}\\ t_{3} \end{array}\right.$$

where $t_{1}=0$, $t_{2}=\sin^{-1}\frac{c_{1}}{d}$, $t_{3}=0$, $t_{4}=\sin^{-1}\frac{c_{1}}{c}$.

#### 2.4.1. Calculating Bending Moment

After free bending deformation, there is a tangential force equilibrium relationship, as shown in Equation (28), and a moment equilibrium relationship, as shown in Equation (29), between the internal stress and external load of the section along the *z*-axis.

(28) $$F_{T}=\underset{A_{1}}{\int }\sigma dA+\underset{A_{2}}{\int }\sigma dA+\underset{A_{3}}{\int }\sigma dA$$

(29) $$M=\underset{A}{\int }\sigma udA=\underset{A_{1}}{\int }\sigma udA+\underset{A_{2}}{\int }\sigma udA+\underset{A_{3}}{\int }\sigma udA$$

Substituting Equation (7) into Equation (28) can obtain the expression of tangential force$\;F_{T}$.

(30) $$\begin{array}{rr} F_{T} & =\underset{A_{1}}{\int }-\sigma_{Y}+\left(\frac{\left(R-\rho_{\varepsilon }\right)-u}{\rho_{\varepsilon }}+\varepsilon_{Y}\right)DdA+\underset{A_{2}}{\int }\frac{\left(R-\rho_{\varepsilon }\right)-u}{\rho_{\varepsilon }}EdA\\ & +\underset{A_{3}}{\int }\sigma_{Y}+\left(\frac{\left(R-\rho_{\varepsilon }\right)-u}{\rho_{\varepsilon }}-\varepsilon_{Y}\right)DdA \end{array}$$

In addition, substituting Equation (7) into Equation (29) can obtain the expression of total bending moment M.

(31) $$\begin{array}{ll} M & =\underset{A_{1}}{\int }\left[-\sigma_{Y}+\left(\frac{\left(R-\rho_{\varepsilon }\right)-u}{\rho_{\varepsilon }}+\varepsilon_{Y}\right)D\right]udA+\underset{A_{2}}{\int }\frac{\left(R-\rho_{\varepsilon }\right)-u}{\rho_{\varepsilon }}EudA\\ & +\underset{A_{3}}{\int }\left[\sigma_{Y}+\left(\frac{\left(R-\rho_{\varepsilon }\right)-u}{\rho_{\varepsilon }}-\varepsilon_{Y}\right)D\right]udA \end{array}$$

#### 2.4.2. Calculating the Position of the Elastic–Plastic Boundary of the Cross-Section and the Radius of the Strain Neutral Layer

Bringing $u=c_{0}$, $u=c_{1}$ and $\varepsilon_{Y}$ into Equation (5), we can calculate the position of the elastic–plastic boundary line of the cross-section of the tube after bending.

(32) $$c_{0}=\left(R-\rho_{\varepsilon }\right)-\rho_{\varepsilon }\varepsilon_{Y}$$

(33) $$c_{1}=\left(R-\rho_{\varepsilon }\right)+\rho_{\varepsilon }\varepsilon_{Y}$$

So far, the analytical expressions for all the variables on the cross-section of the tube during free bending deformation have been given. Moreover, when the free bending radius R is given, by solving Equations (30), (32), and (33) simultaneously, the variable values $c_{0}$, $c_{1}$, and $\rho_{\varepsilon }$ in two elastic–plastic states can be solved. Finally, by taking the variables $c_{0}$, $c_{1}$, and $\rho_{\varepsilon }$ into Equation (31), the moment M on the cross-section required for deformation can be calculated. Next, in order to verify the accuracy and validity of the above analytical model, these analytical formulas were implemented in MATLAB software and compared with the SFEM, FEM, and actual machining results of tube samples to prove that this analytical model is feasible to analyze the bending deformation of tube.

## 3. Finite Element Simulation Model

### 3.1. Finite Element Model of Free Bending Forming

**Figure 6:**
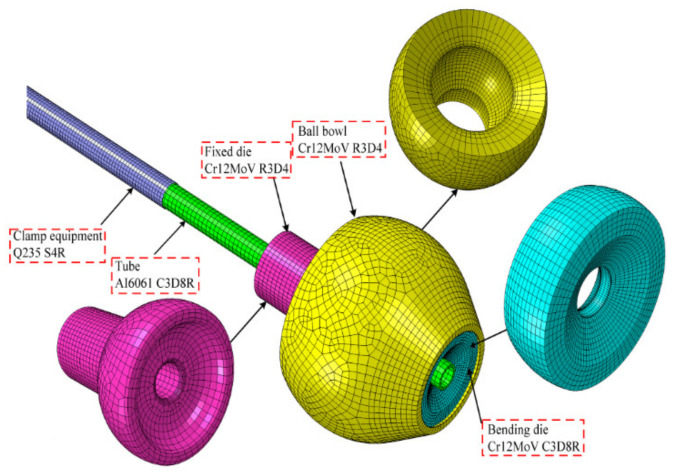
FEM of a 3D spatial free bending system.

The finite element model (FEM) used to verify the correctness and feasibility of the analytical model for the deformation evolution of the cross-section of the tube during the free bending forming process is shown in Figure 6, which was meshed in ABAQUS/Explicit. The finite element mesh size of the tube was 0.8 mm, and the mesh size of each other component was divided according to the size of the tube mesh. This model mainly focuses on the motion behavior and contact conditions between the tube and the bending die. Thus, the bending die and tube were set as deformable solid parts, meshed with 8-node hexahedral linear elements (C3D8R). On the other hand, to reduce the amount of computation, other components such as the ball bowl and the fixed die were set as discrete rigid bodies meshed with 4-node rigid elements (R3D4), and the clamping equipment was set as a shell of 4-node curved shell elements (S4R). The contact form was universal contact, and the friction coefficient was 0.1 and obeyed the Coulomb friction formula. The mass scaling factor was 10,000 times. The Von Mises criterion was adopted.

### 3.2. Mechanical Parameters Used in FEM

**Figure 7:**
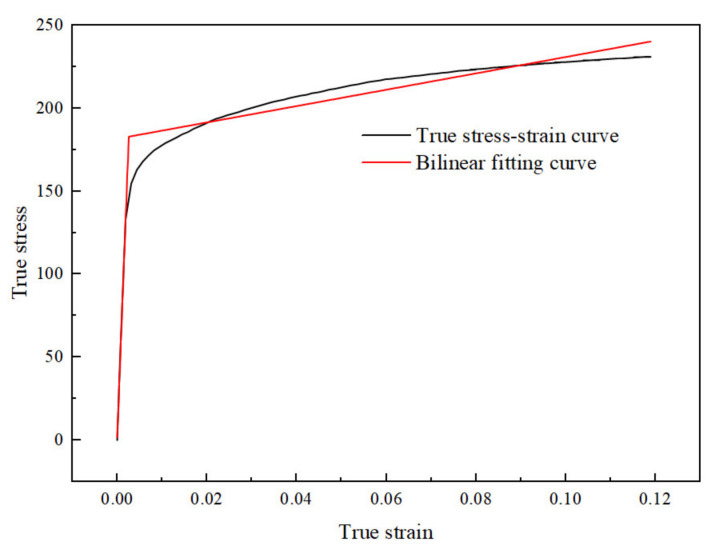
Stress–strain curves.

The materials of the tube and bending die were aluminum alloy and die steel, respectively. Tensile tests complying with GB/T228-2002 were conducted on an AG-Xplns100KN universal testing machine, and the tensile strain rate was 0.5 mm/s. Corresponding mechanical properties and strain–stress curves are shown in Table 1 and Figure 7.

### 3.3. SFEM of Pure Bending Forming

**Figure 8:**
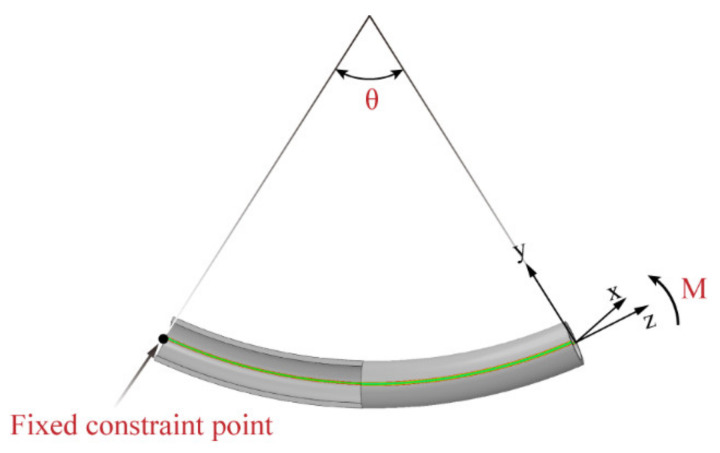
SFEM to simulate bending without tool contact.

To analyze the evolution of stress and strain with bending deformation over the cross-section without the influence of tool contact and contact stresses, as illustrated in Figure 8, SFEM was set up, in which bending was applied by boundary conditions. Since there is no contact interface in this approach, without considering friction and inertia, the calculation results will not be disturbed by changes in external conditions. On the other hand, so as to make the actual tube bending process have static characteristics within a given feed rate range, SFEM was regarded as a static problem, and an implicit solver was used to solve it. The advantage of using this calculation scheme is that the equilibrium equations will be solved in each time step, to maintain static equilibrium in the whole simulation process [28]. This allows a reliable analysis of mathematical constructs that cannot be directly validated by FEM and experiments. Like the explicit model shown in Figure 7, SFEM was also meshed with C3D8R elements. Moreover, to make the simulation conditions closer to the real bending environment of the tube, only a 57 mm long tube segment was considered, which represents the tube length between the center of the bending die hole and the center point of the fixed die outlet, with boundary conditions at both ends of the tube simulating bending.

Here, as illustrated in Figure 8, bending was applied around the binormal vector (the normal vector of the curved plane) at one end of the tube segment by a rotating boundary condition, and a fully fixed constraint was imposed by a fixed boundary condition at the neutral axis at the other end of the segment. The rotation around the binormal vector was given by the bending angle θ, which, in the specific case of a 57 mm long tube segment, was equal to 57 mm/R.

## 4. Performance of the Analytical Model

### 4.1. Bending Tubes by Using the Four-Axis Free Bending Forming Device

#### 4.1.1. Four-Axis Free Bending Forming Device

**Figure 9:**
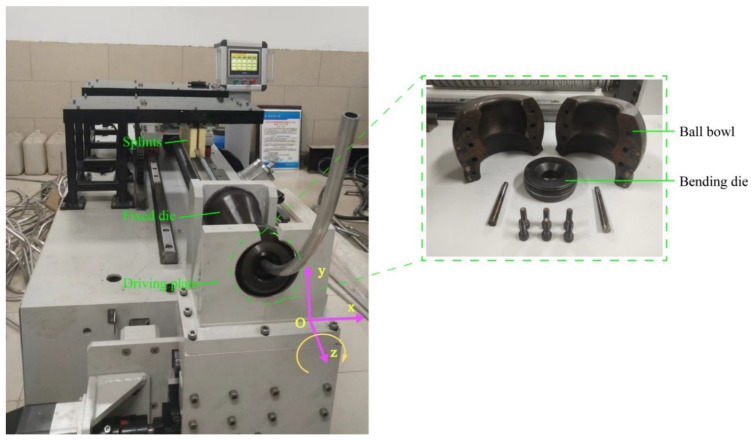
Four-axis free bending forming equipment.

The AI 6061 tube used for finite element analysis and tube processing was a round tube with an outer diameter of 18 mm and an inner diameter of 14 mm. Moreover, the bending of the tube was completed by four-axis free bending equipment as shown in Figure 9, which was independently developed and produced by the team. The bending machine is mainly composed of a rotatable transportation system and a die assembly. The rotatable transportation system consists of a motor outputting thrust, a motor outputting torque, and two pairs of splints. They feed the tube forward into the die assembly (*z*-axis). The die assembly is mainly composed of a fixed die, a ball bowl, a bending die, and a driving plate. Through the driving plate, the bending die leaves the coordinate origin and rotates around the fixed die driven by the *x*-axis motor and *y*-axis motor. The continuous change of the bending die position can make the tube realize continuous gradual radius forming or spiral curve forming. Indeed, by replacing different die components, round, square, or special-shaped tubes can be processed separately.

#### 4.1.2. Process Planning

Firstly, when starting to bend a tube, the driving plate moves away from the coordinate origin to the target position. Its moving distance is

(34) $$H=h+\frac{L\cdot \;\frac{r}{2R}}{\sqrt{1-{\left(\frac{r}{2R}\right)}^{2}}}\;$$

where H denotes the total distance that the motor pushes the driving plate in the *y*-axis direction, h denotes the gap between the driving plate and the initial position of the ball bowl, L represents the horizontal distance between the driving plate and the center point of the fixed die outlet, and r is the radius of rotation of the bending die around the center point of the exit of the fixed die.

Secondly, when the tube is stably formed, the driving plate and the bending die are kept in a fixed position.

Thirdly, when the target tube is completed, the driving plate drives the ball bowl to return to the *z*-axis of the machine tool coordinate. The moving distance of the driving plate is

(35) $$H^{\prime }=-\left|2h+\frac{L\cdot \frac{r}{2R}}{\sqrt{1-{\left(\frac{r}{2R}\right)}^{2}}}\right|$$

Finally, the driving plate returns to the machine coordinate origin. Its moving distance is h.

### 4.2. Verification of Section Deformation Using the Analytical Model

**Figure 10:**
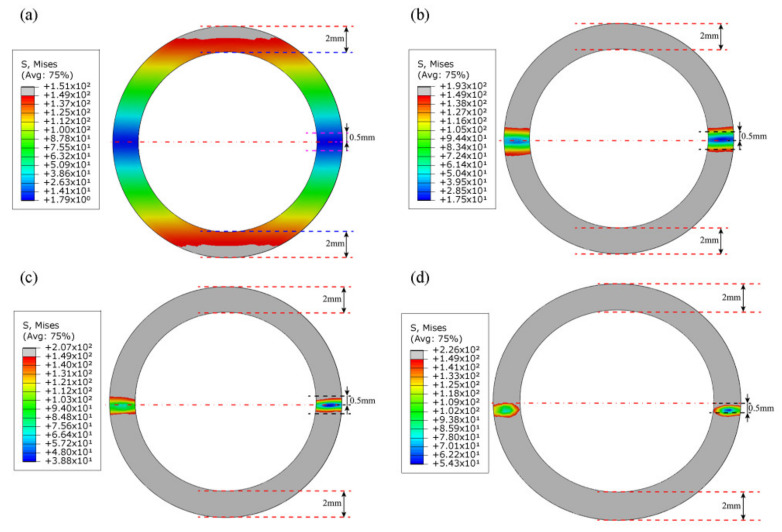
Elastoplastic boundary evolution of SFEM as the bending radius R varies, (**a**) when R=3600 mm, (**b**) when R=400 mm, (**c**) when R=200 mm,  (**d**) when R=100 mm.

Four sets of bending radius data were selected to verify the validity of the analytical model. The first set of data should satisfy Case (a) in Section 2.2 and make the elastic–plastic boundary line shown in Figure 5a, located between c and d, and thus the bending radius R was set to 3600 mm. The result of SFEM is depicted in Figure 10a. Similarly, the second group of data should conform to Case (b) in Section 2.2, and the elastoplastic boundary line depicted in Figure 5b should be located between the inner radius c and the *x*-axis, so the selected bending radius R was set to 400 mm and 200 mm. The results of SFEM are depicted in Figure 10b,c. Furthermore, the third set of data should also conform to Case (b), but the elastoplastic boundary line illustrated in Figure 5b was located under the *x*-axis. Thus, the selected bending radius R was set to 100 mm. The result of SFEM is depicted in Figure 10d. In fact, for Case (b), three bending radii were chosen with radius values of 400 mm, 200 mm, and 100 mm because the actual tube radii were thought to be almost within this range.

Figure 10 shows the evolution of the elastic–plastic boundary with bending radius R in the SFEM. Table 2 demonstrates the data characterization of key parameters in section deformation between the analytical model, SFEM, FEM, and actual sample processing. The bending moments calculated by the analytical model, SFEM, FEM, and actual sample processing are $\mathrm{denoted}\;\mathrm{by}\;M_{1}$, $M_{2}$, $M_{3}$, and $M_{4}$, respectively. When R=3600 mm, as shown in Table 2, according to the analytical formula, $c_{0}$ and $c_{1}$ were calculated as −7.56 mm and 7.56 mm, respectively. This is the same as the result in Figure 10a, except that the values of $c_{0}$ and $c_{1}$ in Figure 10a are close to ±8 mm. This shows that the degree of deformation of the tube section calculated with the analytical model is larger than that calculated by SFEM.

As shown in Table 2, the same conclusion can also be drawn by comparing the calculated bending moment values ($M_{1}=61,196$, $M_{2}=58,016$).

**Figure 11:**
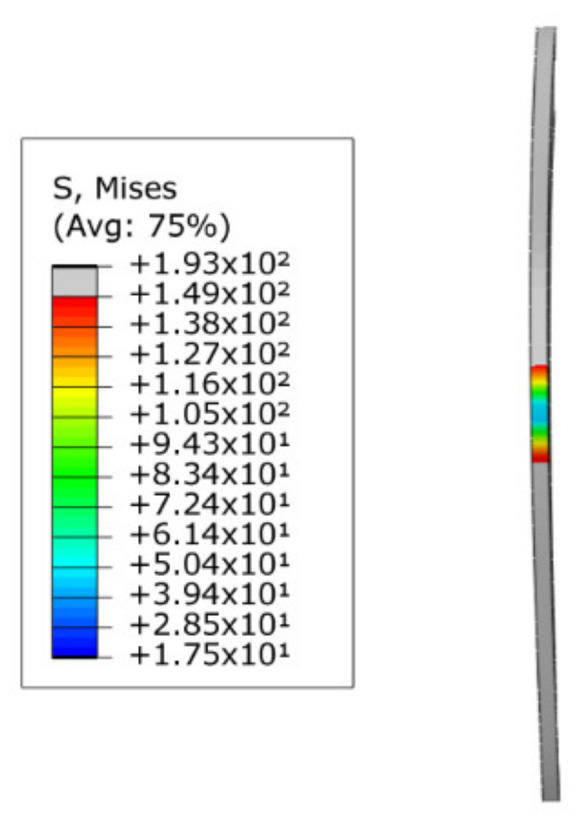
Tube cross-section deformation.

When R=400 mm, the analytical model calculated $c_{0}$ and $c_{1}$ values of −0.839 mm and 0.84 mm, respectively. The difference between the two was 0.001 mm. However, since the difference is too small, the radius $\rho_{\varepsilon }$ of the strain neutral layer coincides with the radius R to the geometric center layer. On the other hand, the values of $c_{0}$ and $c_{1}$ shown in Figure 10b are around ±0.6 mm to ±0.7 mm. This demonstrates that the degree of deformation of the tube section calculated by the analytical model is smaller than that calculated by SFEM. The same conclusion can also be drawn by comparing the bending moment values ($M_{1}=80,131$, $M_{2}=94,547$). This may be due to the energy consumed in the *z*-direction fiber elongation in the tensile deformation zone, the *z*-direction fiber shortening in the compressive deformation zone, and the warpage deformation on the tube section, as shown in Figure 11.

When R=200 mm, the $c_{0}$ and $c_{1}$ calculated by the analytical model were −0.419 mm and 0.42 mm, respectively, and the difference between the two was still 0.001 mm. Likewise, the values of $c_{0}$ and $c_{1}$ shown in Figure 10c are also approximately −0.4 mm and 0.45 mm, respectively. In addition, from Figure 10c, it can be seen that the elastoplastic boundary line of the tensile deformation area is closer to the centerline than the elastoplastic boundary line of the compressive deformation area. This may be attributed to the plastic flow compensation of the y-direction fibers to the *z*-direction fibers in the tensile zone, which promotes the rapid movement of the elastoplastic boundary line to the central layer. In contrast, the compression zone becomes thicker in the y-direction due to the compressive motion of the fibers in the *z*-direction, so that the speed of $c_{1}$ moving towards the center layer lags behind $c_{0}$.

When R=100 mm, in the analytical model, the values of $c_{0}$ and $c_{1}$ continued to decrease, reaching −0.21 mm and 0.21 mm, respectively. Nevertheless, in SFEM, as shown in Figure 10d, there is an obvious displacement of the strain neutral layer, which is approximately 0.4 mm. In addition, the elastic–plastic boundary line of the stretching deformation area is already located below the geometric center layer at this moment, at about 0.1 mm. On the contrary, the elastic–plastic boundary line of the compressive deformation area retreats to 0.6 mm. At the same time, it can also be seen that a certain degree of wall thickness thinning occurs on the convex side. Based on the above analysis, it can be found that in this case, the values of $c_{0}$ and $c_{1}$ between the analytical model and SFEM have a huge deviation. This may be due to the lack of characterization parameters such as wall thickness change and section distortion in the analytical model, and the wall thickness change and section distortion are the main causes of strain neutral layer offset. Hence, when the section deformation is severe, the calculation of $c_{0}$ and $c_{1}$ has a large deviation between the analytical model and SFEM. That is, when R≥200 mm, the variation of $c_{0}$ and $c_{1}$ in the analytical model and SFEM are consistent; when R<200 mm, the calculation results of $c_{0}$ and $c_{1}$ in the two are deviated.

**Figure 12:**
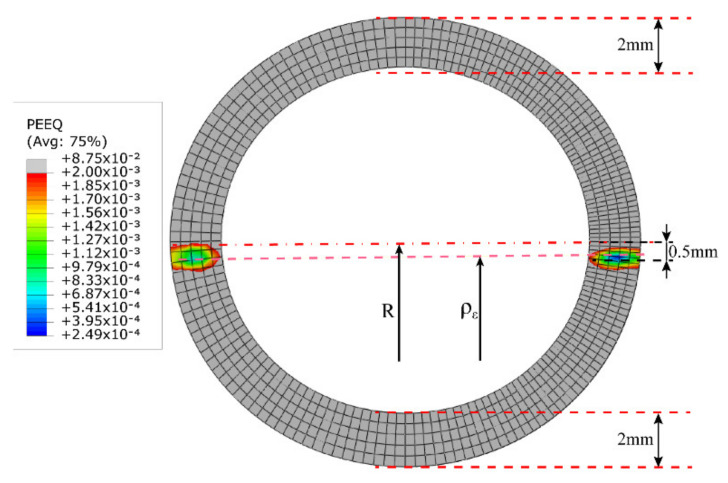
The position of the strain neutral layer $\rho_{\varepsilon }$ shown by SFEM when the bending radius R is 100 mm.

The change law of $\rho_{\varepsilon }$ has the same problem. Since the difference between $\rho_{\varepsilon }$ and R is very small, and the maximum is only 0.001 mm, the offset of the strain neutral layer is not displayed in the analytical model. This results in a large deviation between $\rho_{\varepsilon }$ in the analytical model and SFEM when R decreases to a certain extent, such as 100 mm. As shown in Figure 10d and Figure 12, the stress neutral layer and strain neutral layer have deviated from the geometric center layer relatively significantly and moved towards the compression deformation region, which will prevent the elastoplastic boundary in the compression deformation region from moving further to the geometric center layer. At this time, the position of the neutral layer cannot be represented in the analytical model and needs to be improved in the future.

In conclusion, it can be seen from the above analysis that $c_{0}$ and $c_{1}$ predicted by the analytical model are accurate and effective on the whole. However, the calculation of the predicted value of $\rho_{\varepsilon }$ is conservative. The subsequent addition of the characterization parameters to the analytical model to characterize the wall thickness change may be more accurate for the calculation of $c_{0}$ and $c_{1}$ and $\rho_{\varepsilon }$ in small bending radii.

### 4.3. Comparison of Bending Moments of Tubes

**Figure 13:**
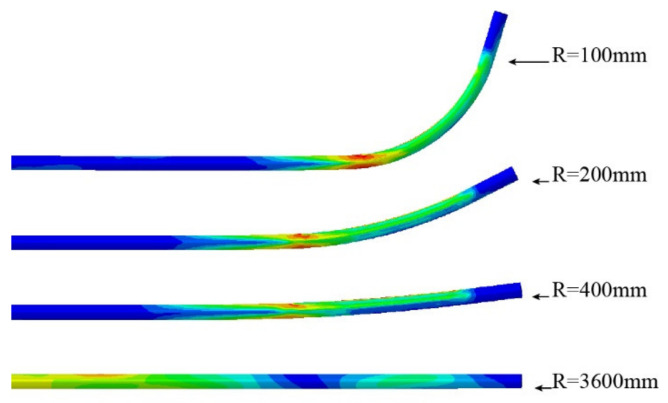
Tube forming in FEM simulation.

**Figure 14:**
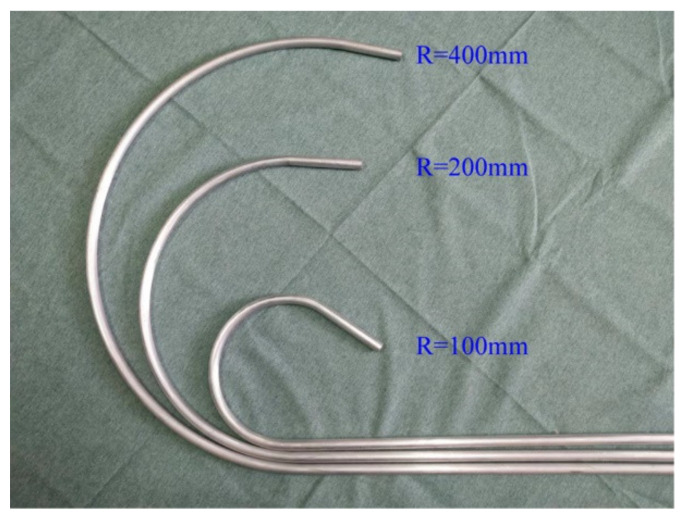
Tube processing in a four-axis free-form bending device.

As described in Table 2, when conducting comparative tests, the analytical model, SFEM, FEM, and experimental investigations must follow the same bending radii, i.e., R=3600 mm, 400 mm, 200 mm, and 100 mm. This requires that, when calculating $M_{3}$ and $M_{4}$, the loading radii should be used in the experiments instead of the radii after springback. If the radii after springback are used for comparison, it can be obtained from the springback law that the loading radii potential must be less than 3600 mm, 400 mm, 200 mm, and 100 mm, respectively. This will inevitably lead to inaccurate comparison results. At the same time, since the springback deformation in free bending is instantaneous springback, the tube shapes seen in FEM and actual experiments are the results of springback, as shown in Figure 13 and Figure 14.

Figure 13 depicts the shapes of tubes with varying radii in FEM. The corresponding bending moments were calculated using Equation (2), which are represented by $M_{3}$ in Table 2. It should be noted that $M_{3}$ is calculated by taking the average of $F_{t}$ and $F_{L}$ values measured in the steady-state region, and the tube advances at a rate of 10 mm/s. Likewise, Figure 14 illustrates the shapes of tubes with varying radii that were completed in a four-axis free-form bending device. The corresponding bending moments were also calculated using Equation (2), which are shown in Table 2 as$\;M_{4}$. It should be noted that $M_{4}$ is computed by averaging the $F_{t}$ and $F_{L}$ values measured in the steady-state region, and the tube moves at a rate of 5 mm/s. In addition, it should be noted that when R=3600, because the bending radius is too large, the eccentric distance U of the bending die is too small. At the same time, the machine tool equipment, particularly the die assembly, contributes to elastic deformation. Consequently, the bending moment cannot be calculated.

$\xi_{i.j}\left(i,j=1,2,3,4\right)\;$was used to express the deviation between the bending moments in Table 2. The deviation between the bending moments can be calculated by Equation (36); the calculation results are shown in Table 3.

(36) $$\xi_{i.j}=\frac{M_{i}-M_{j}}{M_{i}}\cdot 100\%$$

**Figure 15:**
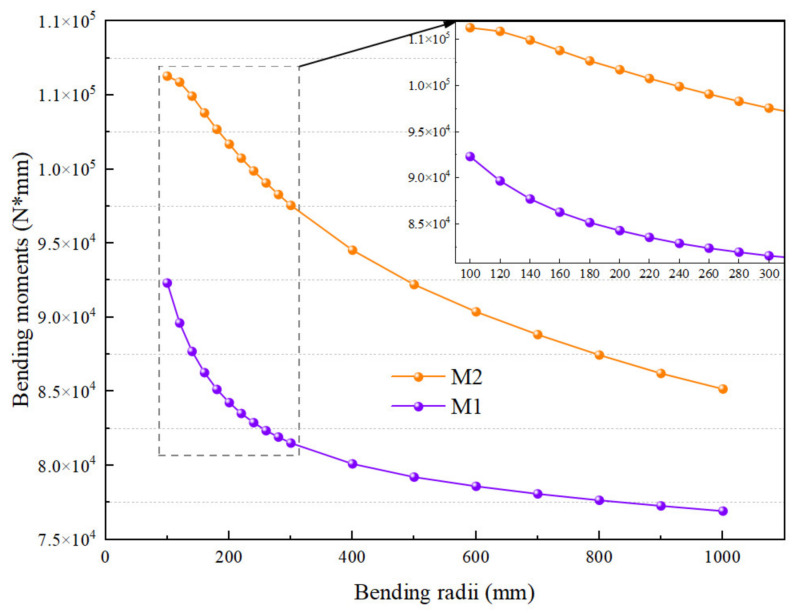
Development trend of bending moment, $M_{1}$ and $M_{2}$, with decreasing radius.

For $\xi_{1.2}$, Table 3 shows that the smallest deviation is 5.19%, which occurs at the bending radius of 3600 mm, when the circular tube has just entered the stage of plastic deformation. In contrast, the largest deviation is −20.71%, which occurs at the bending radius of 200 mm. As shown in Figure 15, comparing $M_{1}$ and $M_{2}$, it is found that the bending moment required for the bending deformation of the tube in SFEM is almost saturated at this time, so the growth of $M_{2}$ is relatively slow.

In contrast, the analytical model does not take into account the influence factors such as wall thickness thinning, wall thickness increase, and cross-section distortion, so $M_{1}$ is still rising rapidly. According to this, it is inferred that $\xi_{1.2}$, $\xi_{1.3}$, and $\xi_{1.4}$ all have a trend of increasing first and then decreasing. Table 2 and Table 3 show that FEM as a whole has the largest calculated bending moment value, resulting in the largest deviation of $\xi_{1.3}$ as well. This may be because FEM not only calculates the energy consumed by the deformation of the tube but also calculates the energy required for the friction between the tube and the mold and between the mold and the mold when calculating the bending moment. At the same time, it is also found that the bending moment $M_{4}$ calculated by the machine tool is slightly smaller than the bending moment $M_{3}$ calculated by FEM. This may be due to the elastic deformation and insufficient rigidity of the die device of the machine tool. Thus, the bending moment $M_{1}$ required for the bending deformation of the tube calculated by the analytical model can meet the actual demand. The accuracy and effectiveness of the analytical model are also demonstrated.

### 4.4. Comparison of Several Typical Analytical Bending Moment Results

**Figure 16:**
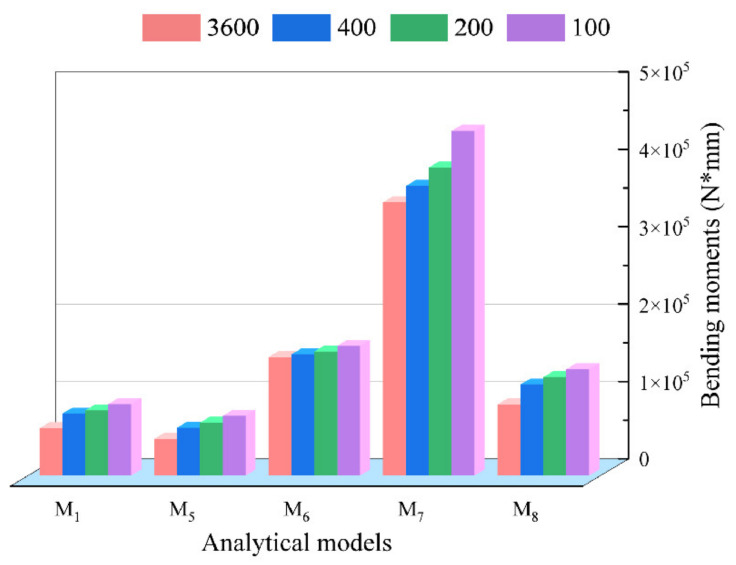
Specific calculation results of $M_{1}$, $M_{5}$, $M_{6}$, $M_{7},$ and $M_{8}$.

In this section, the results and deviations of some typical analytical calculations of tube bending moment are compared.$\;M_{5}$ and $M_{6}$ are used to indicate the bending moments calculated by Lu and Tang, respectively; $M_{7}$ and $M_{8}$ denote the bending moments established by Daxin E [29] using the bilinear material model and exponential material model, respectively. The specific calculation results can be seen in Figure 16.

To more easily characterize the accuracy of each analytical bending moment depicted in Figure 16, the deviation of each analytical bending moment, i.e., $M_{1}$, $M_{5}$, $M_{6}$, $M_{7}$ and $M_{8}$, was calculated by using Equation (36), with $M_{2}$ and $M_{2}$ as the reference benchmarks. The minimum deviation $\delta_{\min}$, maximum deviation $\delta_{\max}$, and average deviation $\delta_{\mathrm{ave}}$ in each group of deviations are shown in Table 4. In addition, $\delta_{\mathrm{ave}}$ is expressed by Equation (37):

(37) $$\delta_{\mathrm{ave}}=\frac{1}{4}\overset{4}{\underset{k=1}{\sum }}\left|\xi_{i.j}\right|$$

where k denotes the sequence number of the radius value in k=1, 2, 3, 4.

For $\xi_{i.2}$ and $\xi_{i.3}$ (i=1, 2, 3, and 4), it can be found in Table 4 that the deviations of $M_{1}$ are almost all the minimum, that is, $\delta_{\min}=5.19\%$ or 2.36%, $\delta_{\max}=-20.71\%$ or −23.13%, $\delta_{\mathrm{ave}}=14.75\%$ or 15.86%. In contrast, although they are all based on the bilinear material model, the deviations of $M_{7}$ are almost all the maximum, that is, $\delta_{\min}=74.44\%$ or 73.92%, $\delta_{\max}=83.56\%$ or 83.07%, $\delta_{\mathrm{ave}}=77.21\%$ or 76.70%. On the other hand, $M_{5}$ is an improvement based on $M_{6}$, so the three deviations of $M_{5}$ are basically better than that of $M_{6}$. Therefore, according to the comparative analysis of deviations, it can be inferred that the order of bending moment accuracy from small to large is $M_{1}<M_{8}$<$M_{5}$<$M_{6}$<$M_{7}$. Thus, based on the above analysis, it can be found that the accuracy of the bending moment calculated by the analytical model provided in this paper is still relatively high, which verifies the accuracy of the analytical model.

## 5. Conclusions

This work focuses on providing insight into the elastic–plastic deformation of the cross-section in tube bending. The main conclusions and remarks are as follows:

(1) An analytical model is developed to accurately clarify the evolution of the elastic–plastic deformation of the cross-section in tube bending, not only qualitatively but also quantitatively, which can calculate the position of the elastic–plastic boundary of the tube and the radius of the strain neutral layer and the applied bending moment on the cross-section under a given bending radius.
(2) The evolution of section elastic–plastic deformation predicted by the analytical model is consistent with SFEM as a whole. As the bending radius gradually changes from large to small, the position of the elastic–plastic boundary line is basically consistent with the SFEM. Only when the radius is reduced to a certain extent, such as 100 mm, there is a certain deviation between the two results.
(3) The bending moments with varying radii calculated by the analytical model are in accordance with the results of SFEM, FEM, and the experimental investigation. Furthermore, compared with Tang’s model, Lu Shiqiang’s model and Daxin’s model, the bending moment errors calculated by this analytical model tend to be much better than the existing model. With the future introduction of wall thickness deformation, it is expected that the deviation of the bending moment, the strain neutral layer, and the elastic–plastic boundary will be reduced even further.

## Figures and Tables

**Table 1:** Mechanical properties used in FEM.

Material	Young’s Modulus E/GPa	Yield Stress $\sigma_{S}/MPa$	Ultimate Strength $\sigma_{B}/MPa$	Poisson’s Ratio μ	Density $\rho /\left(Kg/m^{3}\right)$	LHC D/MPa
AI6061	69.85	149.2	228.2	0.3	2.71 × 10^3^	492
Cr12MoV	218	750		0.28	7.85 × 10^3^	

**Table 2:** Comparison of section deformation parameters between analytical model, SFEM, FEM, and actual sample processing.

R mm	$c_{0}$ mm	$c_{1}\;mm$	$\rho_{\varepsilon }\;mm$	$M_{1}\;N\cdot mm$	$M_{2}$ N·mm	$M_{3}\;N\cdot mm$	$M_{4}$ N·mm
3600	−7.56	7.56	3600	61,196	58,016	59,754	-
400	−0.839	0.84	399.99	80,131	94,547	96,369	95,868
200	−0.419	0.42	199.99	84,258	101,701	103,747	102,629
100	−0.209	0.21	99.99	92,322	106,281	108,653	107,851

**Table 3:** Deviation calculation between bending moments in Table 2 (unit: %).

R mm	$\xi_{1.2}\;$	$\xi_{1.3}\;$	$\xi_{1.4}$	$\xi_{2.3}$	$\xi_{2.4}$	$\xi_{3.4}$
3600	5.19	2.36	-	−2.99	-	-
400	−17.99	−20.26	−19.63	−1.93	−1.38	0.52
200	−20.71	−23.13	−21.80	−2.01	−0.91	1.08
100	−15.12	−17.69	−16.82	−2.23	−1.48	0.74

**Table 4:** Accuracy analysis of bending moments: $M_{1}$, $M_{5}$, $M_{6}$, $M_{7}$ and $M_{8}$ (unit: %).

	$M_{1}$		$M_{5}$		$M_{6}$		$M_{7}$		$M_{8}\;$	
	$\xi_{1.2}\;$	$\xi_{1.3}\;$	$\xi_{5.2}$	$\xi_{5.3}$	$\xi_{6.2}$	$\xi_{6.3}$	$\xi_{7.2}$	$\xi_{7.3}$	$\xi_{8.2}$	$\xi_{8.3}$
$\delta_{\min}$	5.19	2.36	−22.62	−26.29	36.55	35.16	74.44	73.92	19.62	18.08
$\delta_{\max}$	−20.71	−23.13	−53.07	−56.02	62.09	60.96	83.56	83.07	36.89	35.00
$\delta_{\mathrm{ave}}$	14.75	15.86	40.55	43.73	43.70	42.45	77.21	76.70	24.79	23.10

## Data Availability

No new data were created or analyzed in this study. Data sharing is not applicable to this article.

## Nomenclature

R	Bending radius of the original center layer after tube deformation
ρ	Bending radius of bent tube at any position
$R_{i}$, $R_{o}$	Innermost and outermost bending radius of the tube, after bending deformation
$\rho_{\varepsilon }$	Bending radius of strain neutral layer, after tube bending deformation
θ	Bending angle after tube deformation
A	Distance from the center of the bending die to the front end of the guiding mechanism
U	Eccentricity of bending die
λ	Deflection angle of bending die
*c*, *d*, $t_{o}$	Outer radius, inner radius, and wall thickness of the tube
*E*	Young’s modulus
D	Linear hardening coefficient (LHC) of material
$c_{0}$	Distance from the boundary layer between elastic deformation zone and tensile plastic deformation zone to the geometric center layer
$c_{1}$	Distance from the boundary layer between elastic deformation zone and compressive plastic deformation zone to the geometric center layer
s	Relative distance from a point P on the section to the strain neutral layer $\rho_{\varepsilon }$
u	Relative distance from a point P on the section to the geometric center layer R
M	Bending moment applied on the cross-section of tube
$F_{T}$	Tangential force applied on the cross-section of tube
$F_{L}$	Z-axis thrust of propulsion mechanism acting on the tube
$F_{t}$	The force, applied by bending die, perpendicular to the axis of the tube
$\sigma_{i}$, $\sigma_{o}$	Tangential stress of the innermost and outermost fibers of tube
$\varepsilon_{i}$, $\varepsilon_{o}$	Tangential strain of the innermost and outermost fibers of tube
σ, $\sigma_{Y}$, $\sigma_{z}$	Stress, yield stress, tangential stress
ε, $\varepsilon_{Y}$, $\varepsilon_{z}$	Strain, elastic limit strain, tangential strain
